# Anticipated Threat Amplifies Esophageal Perception in the Absence of Peripheral Change

**DOI:** 10.1111/nmo.70503

**Published:** 2026-11-12

**Authors:** Livia Guadagnoli, Jonas Zaman, Jutta Windey, Dimitri van Ryckeghem, Stefaan Van Damme, Lukas Van Oudenhove

**Affiliations:** ^1^ Kenneth C. Griffin Esophageal Center, Department of Gastroenterology and Hepatology Northwestern University Feinberg School of Medicine Chicago Illinois USA; ^2^ Laboratory for Brain‐Gut Axis Studies (LaBGAS), Translational Research Center for Gastrointestinal Disorders (TARGID), Department of Chronic Diseases and Metabolism (CHROMETA) KU Leuven Leuven Belgium; ^3^ REVAL Rehabilitation Research, Faculty of Rehabilitation Sciences UHasselt Hasselt Belgium; ^4^ Quantitative Psychology and Individual Differences, Faculty of Psychology and Educational Sciences KU Leuven Leuven Belgium; ^5^ Department of Clinical Psychological Science Maastricht University Maastricht the Netherlands; ^6^ Department of Experimental‐Clinical and Health Psychology Ghent University Ghent Belgium

**Keywords:** behavioral medicine, esophageal disorders, Psychogastroenterology, symptom perception

## Abstract

**Background:**

Psychological processes may influence how gastrointestinal sensations are perceived and responded to, but their impact on esophageal symptom perception remains poorly understood. We developed and tested a novel stimulus–response paradigm to examine how anticipated threat influences behavioral responding, fear, and perceived intensity of esophageal sensations.

**Methods:**

Healthy volunteers detected and rated neutral electrical stimulations to the distal esophagus under anticipated threat (potential pain) and control (no pain) conditions, pressing a response button (reaction time) and rating intensity and fear after each stimulus. To evaluate specificity, the paradigm was also applied to somatic hand stimulation. Linear mixed‐effects models tested whether threat produced shorter reaction times and heightened fear and intensity ratings, and whether these effects differed by body location.

**Key Results:**

The sample included 46 participants. Participants rated stimuli in the threat condition as more intense and fear‐evoking than control stimuli (both *p* < 0.0001), despite the stimuli being physically identical. Fear ratings were higher for esophageal than hand stimulation (*p* < 0.0001). Contrary to our hypothesis, response times were slower under threat (*p* = 0.0003) and for esophageal versus hand stimulation (*p* < 0.0001). Threat effects across all outcomes did not differ by body location *p*, suggesting a more general effect of threat across visceral and somatic stimulation.

**Conclusions & Inferences:**

Threat amplified perceived intensity and fear in response to neutral esophageal stimulation. Contrary to expectations, response times were slower under threat, suggesting threat may potentially interfere with rather than facilitate behavioral responding. These findings support a role for threat‐related processes in esophageal symptom perception.

## Plain Language Summary

It is hypothesized that psychological processes, such as being worried or afraid of symptoms or paying a lot of attention to the body, influence how patients experience esophageal symptoms. This study tested whether simply expecting a painful sensation can change how a sensation feels. Forty‐six healthy volunteers received mild, non‐painful electrical stimulation in the esophagus under two conditions: one where they were told pain might follow, and one where they knew it would not. Although the stimulation was identical in both conditions, participants rated it as more intense and more fear‐provoking when they expected possible pain, and they were also slower to respond to it. These findings suggest that anticipating pain can shape how esophageal sensations are perceived and felt, even without any change in the physical stimulus. This points to expectation and fear as potential targets for reducing symptom burden in patients with esophageal disorders, although more research is needed to confirm findings and disentangle mechanisms.

## Key Points

- Psychological processes such as anticipating pain and feeling fear are thought to influence how people experience bodily sensations, but their direct role in esophageal symptoms had not been experimentally tested.
- Using a novel experimental task in 46 healthy volunteers, we found that anticipating possible pain made an identical, non‐painful esophageal stimulation feel more intense and more fear‐inducing, and slowed how quickly participants responded to it—even though the stimulation itself never changed.
- These findings suggest that esophageal symptom perception may be shaped not only by what's physically happening in the esophagus, but by how threatening a sensation feels to the person experiencing it—indicating that addressing this threat response could be a useful target for reducing symptom burden, pending confirmation in patient populations.

## Graphical abstract

In healthy volunteers, anticipating pain made identical, non‐painful esophageal stimulation feel more intense and more fear‐provoking, with similar effects for hand stimulation. Threat‐related expectations can therefore amplify esophageal perception without any change in the stimulus, identifying a candidate mechanism for testing in patients with esophageal symptoms.

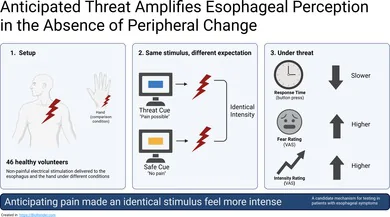

## 1 Introduction

Esophageal symptom perception is characterized by a frequent disconnect between symptom reporting and physiological markers of disease activity. For example, studies evaluating symptom–reflux associations during ambulatory reflux monitoring in patients with gastroesophageal reflux disease (GERD) demonstrate that most reported symptoms are not temporally associated with acid reflux events [1]. Similar discrepancies have been observed in other esophageal conditions, including eosinophilic esophagitis [2, 3] and esophageal motility disorders [4]. One reason for these findings is that esophageal symptom perception comprises both peripheral and central factors, as well as their dynamic interaction along the bidirectional gut–brain axis [5, 6].

Psychological processes, including fear, anxiety, and hypervigilance, are centrally‐mediated factors that contribute significantly to esophageal symptom reporting [4, 7, 8, 9], sometimes above and beyond physiological markers of disease activity [10]. Research indicates that psycho(patho)logical processes are prevalent across different conditions, including GERD, eosinophilic esophagitis, and achalasia, as well as esophageal disorders of gut‐brain interaction, like functional heartburn and functional dysphagia [4, 9, 11]. The Psychobiological Model of Disorders of Gut–Brain Interaction [12] provides a clinically relevant framework for understanding how psychological processes interact with each other and peripheral biology to influence symptom perception.

The model describes a pathway comprising three interrelated processes (Figure 1) [12]: (1) *heightened threat appraisal* of bodily sensations, (2) *fear* as an emotional response to perceived threat, and (3) *interoceptive threat monitoring*, defined as increased attention toward internal sensations [12]. The model posits that within this threat‐fear‐interoceptive threat monitoring pathway, gastrointestinal sensations perceived as threatening elicit fear; this fear, combined with a motivation to reduce uncertainty, increases interoceptive threat monitoring and ultimately amplifies symptom perception [12]. For example, an esophageal patient may interpret a sensation as threatening (e.g., “this heartburn means something is seriously wrong with my esophagus”), experience fear, and focus attention on the esophagus to monitor or control the symptom. This process is hypothesized to heighten esophageal perception, which in turn reinforces the cycle of threat appraisal, fear, and attentional monitoring (e.g., “I feel something, it must be serious, I should be afraid, and I need to keep checking”). However, prior support for this model has come largely from correlational, self‐report data, which cannot establish the direct, mechanistic relationships it proposes.

**FIGURE 1:**
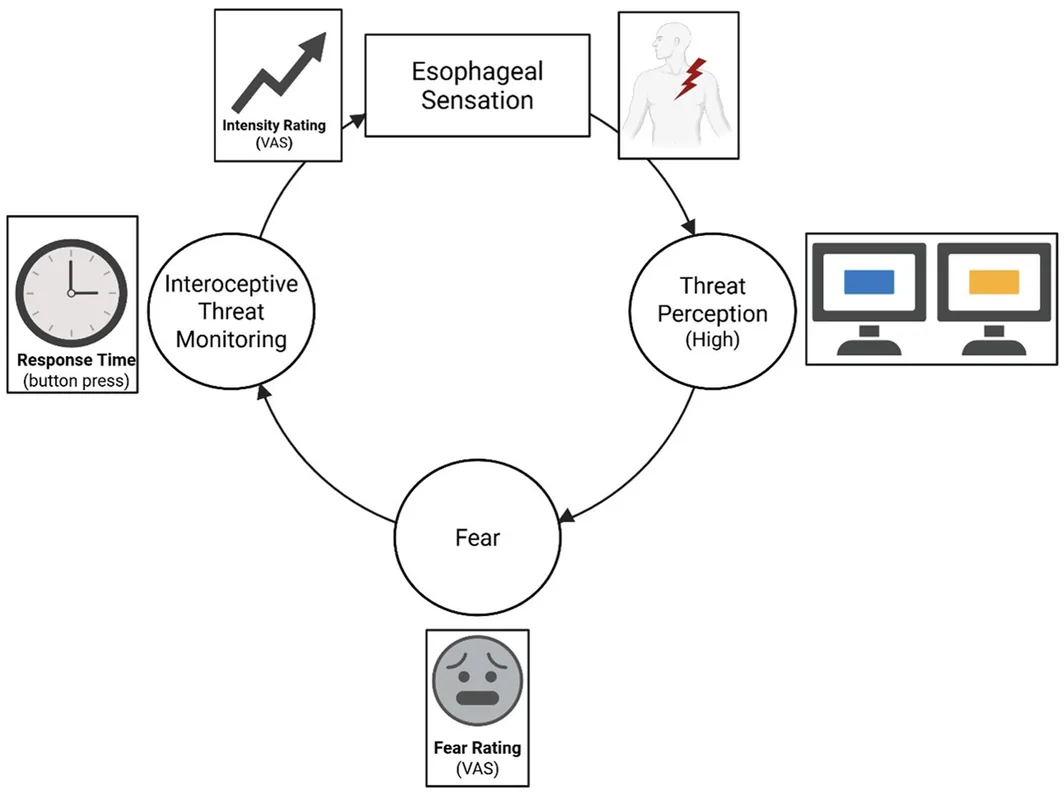
Proposed threat–fear–interoceptive threat monitoring pathway informed by the psychobiological model, and correspondence with experimental paradigm components. The figure illustrates the hypothesized mechanisms proposed by the Psychobiological Model of Disorders of Gut–Brain Interaction [12]. Perceived threat is proposed to evoke fear, which in turn promotes increased interoceptive threat monitoring, ultimately amplifying symptom perception. The experimental paradigm was designed to map onto each of these processes: Threat was manipulated via anticipatory cues signaling potential pain, fear was indexed using trial‐by‐trial fear ratings, behavioral responding to the stimulus was indexed using reaction time, and symptom perception was indexed using intensity ratings of innocuous esophageal stimulation. Created in BioRender. Guadagnoli, L. (2026) https://BioRender.com/2857opl.

Guided by this model, we designed and tested a novel experimental paradigm that evaluates how manipulating threat influences behavioral responding, fear, and perceived intensity of esophageal sensations. Specifically, participants rated the intensity of innocuous electrical stimulations in the esophagus under two conditions: a perceived threat condition, in which they anticipated the possibility of pain, and a control condition, in which they knew no painful stimulation would occur. We also measured participants' fear ratings as well as their reaction time (via a button press) to the stimulation. Reaction time has been used in prior research as a behavioral proxy for attentional engagement with threat [13], on the assumption that faster responding reflects heightened vigilance toward threatening stimuli. To investigate whether these processes were specific to sensations in the esophagus, we conducted the same procedure using the hand as a comparator body location.

Our primary aim was to determine whether anticipation of threat to the esophagus influences fear, behavioral responding, and esophageal perception, consistent with the threat–fear–interoceptive monitoring cycle proposed by the model [12]. We hypothesized that threat anticipation would be associated with heightened fear, increased behavioral responding (i.e., faster reaction time), and greater esophageal intensity perception. We further hypothesized that these effects would be stronger for esophageal compared to hand stimulations, reflecting the higher perceived threat of visceral sensations [14]. In addition, we conducted exploratory analyses to evaluate potential mediators and moderators of the relationship between perceived threat and symptom perception.

## 2 Methods

The study visit consisted of two components: (1) pre‐experimental tasks, which included participant recruitment and enrollment, placement of electrical stimulation and physiological recording equipment, and the collection of demographic, clinical, and psychological information, and (2) the experimental procedure.

### 2.1 Pre‐Experimental Tasks

#### 2.1.1 Participant Recruitment and Enrollment

Healthy adult (Age 18–50) volunteers were recruited through the KU Leuven Faculty of Psychology and Educational Sciences' participant pool management software (Sona Systems; https://www.sona‐systems.com) and posted flyers around the medical campus. Individuals with a history or current state of gastrointestinal, cardiovascular, respiratory, neurological, chronic pain, and psychiatric disorders, and/or abdominal or thoracic surgery (except appendectomy or cholecystectomy) were excluded. Additionally, individuals reporting current pain symptoms (including acid reflux/chest pain), moderate to severe symptoms of anxiety or depression (Patient Health Questionniare‐9 item [PHQ‐9] [15] and/or generalized anxiety disorder‐7 item [GAD‐7] [16] ≥ 10), severe hearing or visual problems, and/or were taking medication with an effect on the visceral and/or central nervous system were excluded. Eligible participants fasted (no food, caffeine, or liquids except water) for at least 6 h before the study visit. The study was approved by the local Ethics Committee Research UZ/KU Leuven (S66372).

#### 2.1.2 Placement of Esophageal and Hand Stimulation

For visceral stimulation, a flexible 2.6 mm catheter (Gaeltec CTO/L‐2E, Isle of Skye, Scotland, UK) with two ring electrodes 8 cm from the tip was placed transnasally, positioning the electrodes at the distal esophagus (~35 cm from the nostril); a 15‐min rest period followed catheter insertion to allow habituation and physiological recovery [17]. For somatic stimulation, a bar electrode (Digitimer, Hertfordshire, UK) with two 8 mm electrodes was attached to the wrist of the nondominant arm via wristband. Electrical stimulations (train of five 500 μs square‐wave pulses, 200 Hz) were delivered to both sites using a constant current stimulator (DS7, Digitimer Ltd), following procedures established in prior work [14].

#### 2.1.3 Demographic, Clinical, and Psychological Information

Participants provided demographic information (age, gender, race) and completed questionnaires via REDCap [18, 19]: the esophageal hypervigilance and anxiety scale (EHAS) [20], intolerance of uncertainty scale (IUS‐12) [21], and fear of pain questionnaire (FPQ‐III) [22]. See Supplement for details.

#### 2.1.4 Physiological Measures

##### 2.1.4.1 Cardiac Electrical Activity

Cardiac activity was monitored throughout via three ECG electrodes (BIOPAC EL503) placed below the left and right clavicle and on the lower left abdomen, connected to an isolated bioamplifier (BIOPAC Systems Inc.) for safety given the proximity of electrical stimulation to the heart.

##### 2.1.4.2 Skin Conductance Response

Skin conductance response (SCR), an index of sympathetic arousal [23], was recorded via two electrodes (BIOPAC EL507) on the hypothenar eminence of the nondominant hand to evaluate emotional arousal to threat versus control cues. See Supplement for recording, analysis, and responder/non‐responder criteria.

### 2.2 Experimental Procedure

The procedure was administered using PsychoPy Builder (v2022.1.3) and consisted of baseline recording, a thresholding procedure, and the stimulus–response paradigm (2 practice blocks of 14 trials; 4 experimental blocks of 104 trials), with 2‐min breaks between components and blocks.

#### 2.2.1 Baseline Recording

A 5‐min baseline recording (participants fixating on a crosshair, minimizing movement) ensured signal quality and safety.

#### 2.2.2 Thresholding Procedure

The stimulus–response task required determining both a non‐painful and painful stimulation for the esophagus and the hand. Non‐painful and painful stimulation intensities were determined separately for the esophagus and hand using a modified ascending method of limits [24] (see Supplement). Participants rated each stimulus on a 9‐point categorical scale (no sensation—faint sensation—sensation—faint discomfort—discomfort—faint pain—pain, yet tolerable—strong pain—most intense pain imaginable); the non‐painful intensity was the highest rated “sensation,” and the painful intensity the highest rated “pain, yet tolerable.” Participants who did not reach a rating of “pain, yet tolerable” for either stimulation despite reaching the maximum permissible intensity (60 mA for the esophagus, a ceiling set for participant safety; 99 mA for the hand, the maximum output of the stimulator device) were classified as hyposensitive to visceral stimulation and excluded from the study. Body location order was counterbalanced (Group A: hand then esophagus; Group B: esophagus then hand).

#### 2.2.3 Stimulus–Response Paradigm

In this novel paradigm (Figure 2), participants responded to stimulation delivered to the esophagus or hand under anticipated threat (possible pain) and control (no pain) conditions, maintaining gaze on a central fixation cross with the index finger of their dominant hand on a response button.

**FIGURE 2:**
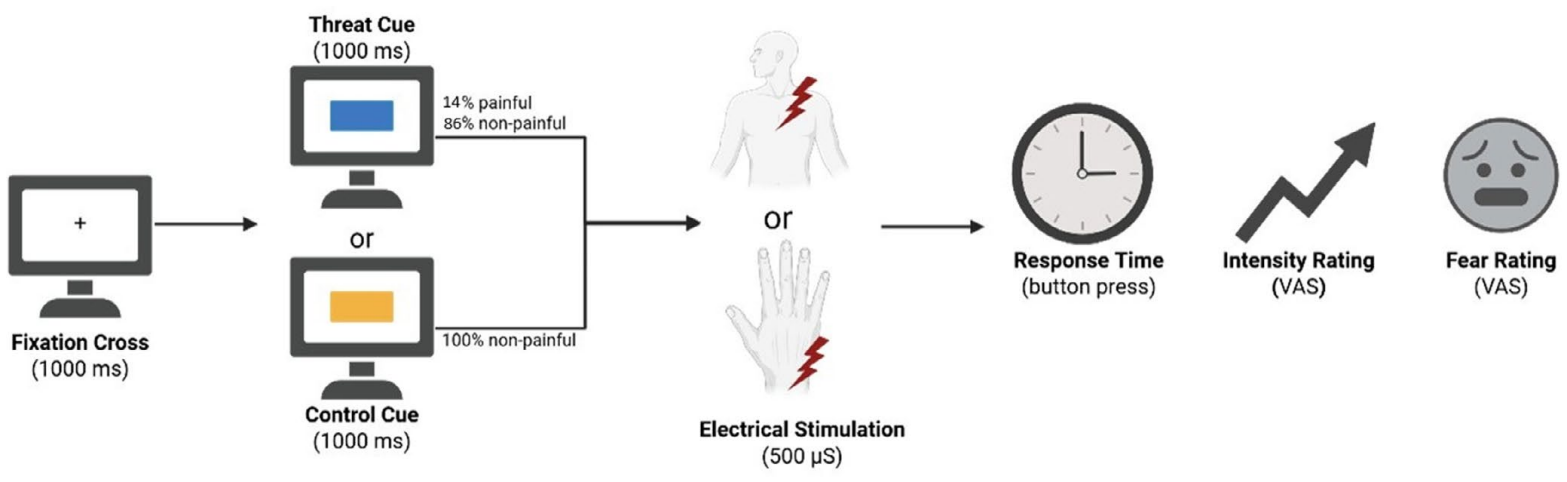
Overview of the Stimulus–Response Paradigm. Participants received electrical stimulations to either the esophagus or the hand under two cue conditions: Threat (potentially painful) and control (non‐painful). Non‐painful stimuli were delivered in the majority of threat trials (86%) and all control trials, with painful stimuli delivered infrequently (14%) in threat trials. Each trial began with a fixation cross followed by a visual cue signaling the upcoming stimulation. Participants responded as quickly as possible upon detecting the stimulus (reaction time), then rated the perceived intensity and fear associated with the cue. Trials were organized into blocks by body location and counterbalanced across participants. Created in BioRender. Guadagnoli, L. (2026) https://BioRender.com/a1ft9ck.

Each trial began with a fixation cross (1000 ms) followed by a visual cue (blue or yellow rectangle; 1000 ms) signaling the upcoming stimulation (500 μs), then the stimulation itself. A blue cue signaled a threat trial (possible painful stimulus); a yellow cue signaled a control trial (non‐painful stimulus guaranteed). Cue color was not counterbalanced across participants. The blue cue consistently signaled threat trials and the yellow cue consistently signaled control trials for all participants. In practice, the non‐painful stimulus was delivered on 86% of threat trials (12/14) and 100% of control trials (12/12); the painful stimulus was delivered on the remaining 14% of threat trials. This low reinforcement ratio was chosen to limit the strength and frequency of cue‐pain associations while still maintaining the credibility of the threat cue, consistent with reinforcement schedules used in other threat‐anticipation paradigms [25, 26].

Primary outcomes were reaction time, fear ratings, and intensity ratings. Participants were instructed to press the response button as soon as they felt the stimulation. Participants then rated stimulus intensity (modified Sensation and Pain Rating Scale [27] [SPARS]; −50 = “no sensation” to +50 = “most intense pain imaginable”) and fear of the visual cue (“how fearful were you of the cue?”; 100‐point VAS, 0–100). Participants were informed beforehand that they had 5 s to respond, whether via button press (detecting the stimulus) or scale rating; the button press or scale disappeared once a response was made or after 5 s elapsed, ending the trial. Trials without a response, including a button press and/or rating, within this window (1.86%) were excluded from analysis.

Practice blocks (one per body location) each comprised 14 trials (6 control, 8 threat [6 no‐pain, 2 pain]); the four experimental blocks (two per location) each comprised 26 trials (12 control, 14 threat [12 no‐pain, 2 pain]). Block order alternated by body location and was counterbalanced (Group A began with esophagus; Group B with hand), with participants informed of the upcoming location before each block. An intensity check preceded each block to control for sensitization/habituation: participants rated one non‐painful and one painful stimulus (order randomized) on the 9‐point scale, with intensities adjusted as needed (see Supplement). Participants were blinded to study hypotheses; experimenters were not blinded to condition, as the study could not be feasibly conducted otherwise.

### 2.3 Statistical Analysis

Statistical analyses were completed in SAS Enterprise Guide (version 8.1). Descriptive statistics (mean [SD] and frequency) were applied to evaluate self‐reported demographic and clinical information, where appropriate. In addition, we evaluated the distribution of data for reaction time, fear, and intensity to identify the need for Box‐Cox transformations to normalize the distribution of the residuals of subsequent models. Practice trials and trials involving painful stimulation were excluded, such that analyses of the threat and control conditions included only the non‐painful stimulations. SAS code and de‐identified data for analyses are available at https://github.com/labgas/proj_threat_esophagus.

#### 2.3.1 Primary Analyses

The primary aim of the study was to examine the effect of the threat condition on the three outcomes: reaction time, fear ratings, and intensity perception. We hypothesized that (1) trials in the threat condition would result in faster responses (i.e., shorter reaction times) and higher fear and intensity ratings compared to control trials, (2) esophageal stimulation would elicit faster responses and higher ratings than hand stimulation, and (3) the effect of the threat condition might vary by body location, such that differences in outcomes between threat and control conditions are greater for the esophagus compared to the hand. To test these hypotheses, we fit linear mixed‐effects models with fixed effects of condition (threat vs. control), body location (esophagus vs. hand), and their interaction (condition by location). Each model also included a random intercept for participant to account for individual differences, and a random effect of block nested within participant.

#### 2.3.2 Exploratory Analyses

We conducted exploratory mediation and moderation analyses to further assess relationships between our variables as well as exploratory analyses to assess psychophysiological responses (i.e., skin conductance response) to threat. Theses analyses were restricted to the esophageal body location, as this was the primary body location of interest. Mediation and moderation analyses were performed using the MEMORE macro (version 2.1) for SAS [28, 29]. See Supplement for further details on the methods for the exploratory analyses.

## 3 Results

### 3.1 Participants

A priori power analysis revealed a sample size of 46 participants provides approximately 90% power to detect a small‐to‐medium condition by location interaction (Cohen's *f* = 0.20) at a significance level of *α* = 0.05, assuming moderate correlations between repeated measures. Exploratory analyses, such as mediation and moderation, may therefore be underpowered.

Sixty‐one participants were recruited for the study. Fifteen participants were excluded for the following reasons: hyposensitivity to visceral stimulation (*n* = 7 [11%]), intolerance of esophageal catheter (*n* = 6 [10%]), and equipment issues (*n* = 2 [3%]). The final sample size included 46 healthy volunteers who were young (mean age = 22.7 [3.4]), mostly female (63%) and mostly White (71%). Consistent with the study's health volunteer eligibility criteria, baseline anxiety and depressive scores (GAD‐7, PHQ‐9) were low. Full participant demographics and responses to baseline questionnaires are provided in Table 1.

**TABLE 1:** Demographic and clinical characteristics of the study sample.

Variable (*N* = 46)	Mean (SD) or frequency (%)
Age	22.7 (3.4)
Gender	
Female	29 (63%)
Male	16 (35%)
Non‐binary	1 (2%)
Race	
Asian	8 (18%)
Black	0 (0%)
White	32 (71%)
Other, or mixed	5 (11%)
Baseline measures	
GAD‐7	2.2 (2.0)
PHQ‐9	2.2 (2.3)
FPQ‐III	70.4 (16.3)
EHAS	16.9 (11.1)
IUS‐12	29.5 (7.5)

Abbreviations: EHAS, esophageal hypervigilance and anxiety scale [20]; FPQ‐III, fear of pain questionnaire [22]; GAD‐7, generalized anxiety disorder‐7 item [16]; IUS‐12, intolerance of uncertainty scale [21]; PHQ‐9, patient health questionnaire‐ 9‐item [15].

### 3.2 Effect of Threat on Reaction Time, Intensity, and Fear

The primary aim of the study was to evaluate the effect of the threat condition (compared to the control condition) on reaction time, fear ratings, and intensity perception.

#### 3.2.1 Reaction Time

Contrary to our hypotheses, participants responded significantly slower to stimulations in the threat compared to control condition, and to stimulations in the esophagus compared to hand [condition: (F(1, 2303) = 13.22, *p* = 0.0003); location: (F(1, 167) = 108.55, *p* < 0.0001)] (Figure 3). The effect of threat on reaction time did not differ by body location (condition by location interaction: F(1, 2327) = 0.77, *p* = 0.38).

**FIGURE 3:**
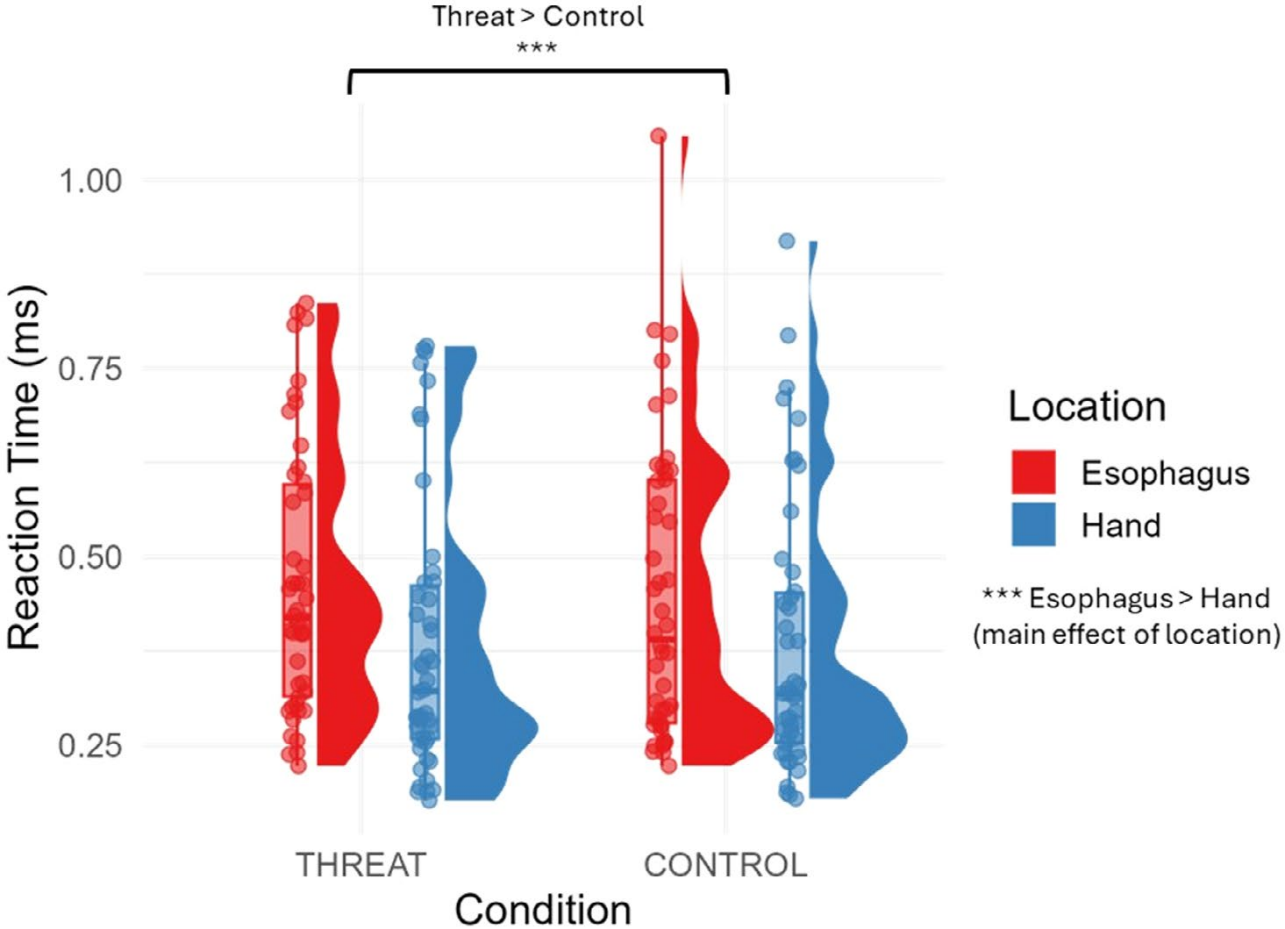
Reaction times by condition and body location. Mean reaction times (ms) are shown for each condition (Control vs. Threat) and body location (Hand, Esophagus). Error bars represent ±1 standard error of the mean. Statistical analyses were conducted on Box‐Cox–transformed values using linear mixed‐effects models; raw values are presented here for interpretability.***p < 0.0003.

#### 3.2.2 Fear

Consistent with our hypothesis, participants reported significantly higher fear ratings in the threat compared to control condition, and to the esophagus compared to hand [condition: (F(1, 3692) = 3749.71, *p* < 0.0001); location: (F(1, 127) = 28.67, *p* < 0.0001)] (Figure 4). As with reaction time, this threat effect was comparable across body locations (condition by location interaction: F(1, 3455) = 0.35, *p* = 0.56).

**FIGURE 4:**
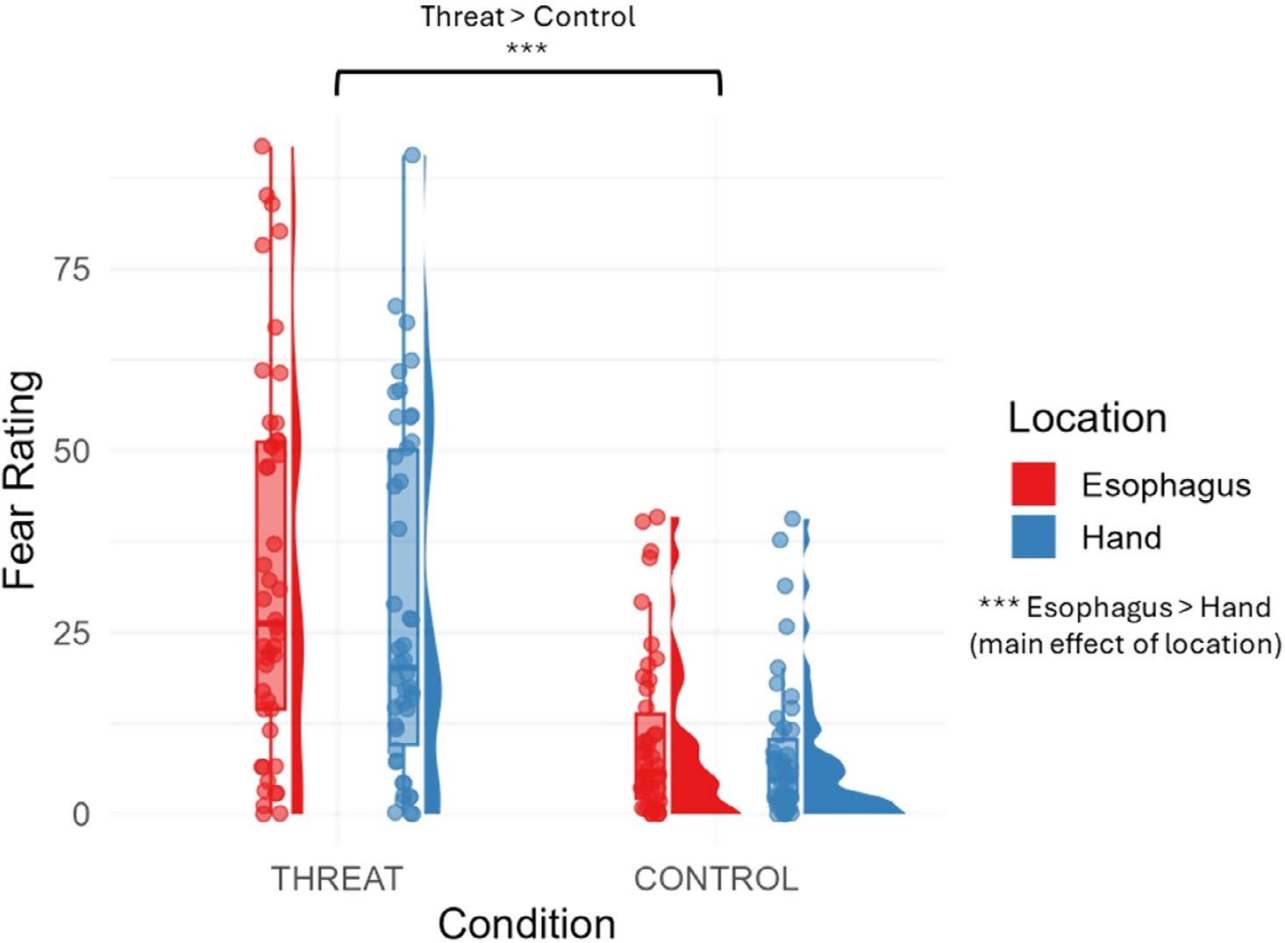
Fear ratings by condition and body location. Mean fear ratings (0–100 scale) are shown for each condition (Control vs. Threat) and body location (Hand, Esophagus). Statistical analyses were conducted on Box‐Cox–transformed values using linear mixed‐effects models; raw values are presented here for interpretability. ***p < 0.0001.

#### 3.2.3 Intensity

Consistent with our hypothesis, participants rated stimulations in the threat condition as significantly more intense compared to stimulations in the control condition, despite the stimulations being set at the same (non‐painful) intensity level (F(1, 2546) = 287.86, *p* < 0.0001) (Figure 5). Intensity ratings were similar between the esophageal and hand locations (F(1, 105) = 0.95, *p* = 0.33), and the condition by location interaction was not significant (F(1, 2489) = 3.50, *p* = 0.06), indicating that the threat‐related increase in intensity did not differ by body location. Descriptively, the numerical increase in intensity under threat was somewhat larger for the esophageal (Δ = 0.36, SE = 0.03, *p* < 0.0001) than hand stimulation (Δ = 0.29, SE = 0.03, *p* < 0.0001), though this difference should be interpreted with caution given the non‐significant interaction.

**FIGURE 5:**
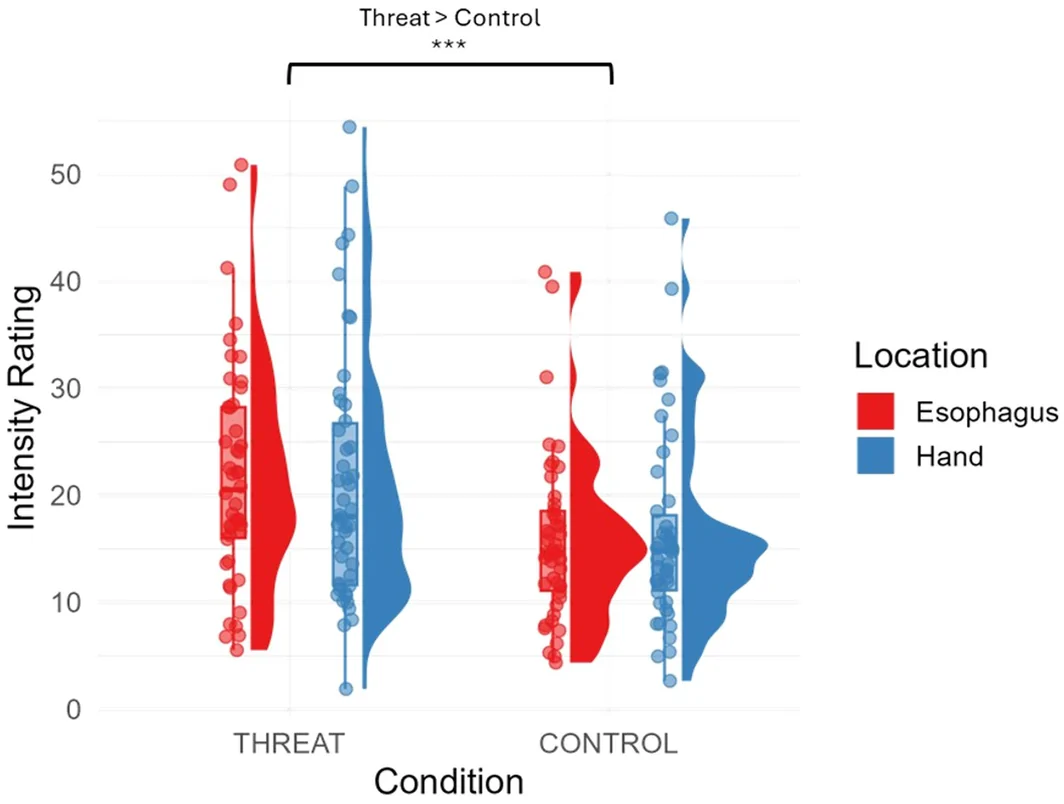
Intensity ratings by condition and body location. Mean intensity ratings (0–100 scale) are shown for each condition (Control vs. Threat) and body location (Hand, Esophagus). Statistical analyses were conducted on Box‐Cox–transformed values using linear mixed‐effects models; raw values are presented here for interpretability. ***p < 0.0001.

### 3.3 Exploratory Analyses

#### 3.3.1 Skin Conductance Response

Fifteen participants were excluded due to either being non‐responders (*N* = 13), missing measurements (e.g., equipment malfunction; *N* = 1), or excessive artifacts that prevented reliable analysis (*N* = 1). Therefore, the final sample included 31 participants. Skin conductance responses, a measure of physiological arousal, were greater during the threat condition compared to the control condition (*β* = 0.078, SE = 0.021, *p* < 0.001), indicating that participants were more physiologically aroused when anticipating potential pain. This pattern was similar for esophageal and hand stimulation (*β* = 0.013, SE = 0.021, *p* = 0.52; interaction *β* = 0.015, SE = 0.021, *p* = 0.46). Responses also tended to decrease across the session (*β* = −0.079, SE = 0.041, *p* = 0.05), consistent with the habituation typically seen with skin conductance measures over repeated trials.

#### 3.3.2 Relationships Between Reaction Time, Fear, and Intensity

We used mediation analyses to test whether fear or reaction time helped explain the relationship between threat and esophageal intensity ratings. For example, does threat lead to increased fear, which then leads to increased intensity ratings? As expected, threat increased both fear ratings and intensity ratings. However, neither fear (indirect effect 95% CI [−0.27, 5.39]) nor reaction time (indirect effect 95% CI [−0.74, 0.20]) explained the relationship between threat and intensity (Supplemental Figures 1 and 2).

We used moderation analyses to test whether individual differences in baseline esophageal hypervigilance and anxiety, intolerance of uncertainty, or fear of pain changed the size of the threat effect on intensity or fear ratings. For example, do people with greater fear of pain show a larger jump in intensity or fear ratings when threat is anticipated? None of these traits moderated the effect of threat on either outcome. Two traits were, however, related to overall fear levels regardless of the condition; participants with greater intolerance of uncertainty reported more fear during control trials (*p* < 0.001) and participants with greater fear of pain reported more fear during both threat and control trials (*p* = 0.05 and *p* = 0.03, respectively). Full statistical results for skin conductance, mediation, and moderation analyses are in Supplement.

## 4 Discussion

This study developed a novel experimental paradigm to examine how anticipated threat influences behavioral responding (i.e., reaction time), fear, and perceived intensity of esophageal sensations. Our primary finding was that participants rated electrical stimulations in the threat condition as significantly more intense than those in the control condition, despite the stimulations being delivered at identical, non‐painful levels. Critically, the only difference between the stimulations was participants' expectation of threat: stimuli following the threat cue (blue rectangle) carried the possibility of pain, whereas stimuli following the control cue (yellow rectangle) did not. Similar effects have been demonstrated in somatic pain research, with a meta‐analysis spanning experimental and clinical studies showing that greater threat appraisals are associated with increased pain perception [30]. Importantly, the present findings extend prior work in esophageal symptom perception by demonstrating that anticipatory threat alone, independent of changes in peripheral input, may amplify visceral perception. This provides initial experimental support for a core assumption regarding the role of threat appraisal in esophageal symptom perception that has previously been inferred largely from correlational self‐report data.

Beyond this central finding, the anticipation of threat also produced broader emotional, behavioral, and psychophysiological changes. Specifically, threat cues were associated with greater fear ratings and increased response time (i.e., slowed responding). In addition, skin conductance response was elevated during threat trials, indicating increased sympathetic arousal. Notably, these threat effects were similar regardless of whether the sensation occurred in the esophagus or the hand. In other words, anticipating a painful sensation appears to amplify perception broadly, rather than being a phenomenon unique to visceral sensations.

One of the primary aims of this study was to examine whether threat would lead to faster reaction times, which we hypothesized would serve a proxy for increased attentional engagement. Contrary to this hypothesis, participants responded more slowly to stimulations in the threat compared to the control condition. There are several possible explanations for our findings, though our reaction time measure alone cannot adjudicate between them. First, attention is multidimensional and includes processes related to orienting toward, engaging with, and disengaging from threatening stimuli [31, 32]. One possible interpretation, consistent with this framework, is that slower reaction times represent difficulty disengaging attention from threat rather than reduced attention per se. That is, threat may prolong attentional engagement with bodily sensations and delay behavioral responding, consistent with the notion that interoceptive threat monitoring reflects sustained monitoring of bodily sensations rather than enhanced speed of detection. This interpretation would be broadly consistent with recent work demonstrating that highly threatening stimuli impair inhibitory performance specifically in individuals who perceive threat as intense, whereas mildly threatening cues may enhance performance [33], as well as other research in somatic pain reporting impaired disengagement from threat cues [34, 35]. Another potential interpretation is that the threat cue may have elicited enhanced cognitive and affective processing (e.g., heightened anxiety), which could have interfered with task performance independent of attentional disengagement per se. Prior work in anxiety disorders has demonstrated slower responses to threat‐related stimuli [36, 37], suggesting that anxiety may inhibit response efficiency even while increasing awareness of threat [36, 38].

Although these interpretations provide a plausible account of the observed slowed responding, it is important to consider our findings within the broader attentional bias literature and the methodological constraints of our paradigm. Prior research in somatic pain has demonstrated threat‐induced attentional biases toward pain‐related stimuli, such as images [39] and words [40, 41]. Importantly, much of this work has been conducted in patient samples, whereas our study examined healthy volunteers, limiting direct comparisons. However, a key difference in our paradigm is that attention was directed toward the body itself rather than an external cue, which increases ecological validity but limits the precision of available attentional measures. Indeed, reaction time reflects a complex combination of cognitive, emotion, and motor processes [42], and may be less sensitive or specific as an index of attentional allocation than measures used in other studies with external cues, such as eye tracking [39, 41]. Future studies could incorporate additional, complementary, indices to help further quantify threat‐induced shifts in attention to bodily sensations, such as (neuro)physiological markers of attention (e.g., pupil dilation, EEG).

Our exploratory analyses aimed to further evaluate potential mediators and moderators of the effect of threat on symptom perception. We examined whether fear and/or reaction time mediated the relationship between threat and symptom perception. Similarly, we assessed whether baseline psychological symptoms moderated the effect of threat on intensity or fear ratings. We did not find any significant mediation or moderation effects. One explanation for these null findings is limited statistical power. For both mediation and moderation analyses, we averaged across all trials, which reduced the number of observations per participant in our sample of 46 individuals. Additionally, for the moderation analyses, limited variability in baseline measures, due to the sample being comprised of healthy volunteers, may have further limited our ability to detect moderating effects (Supplemental Figures 3 and 4). A next step would be to assess this task in a larger sample and a patient population to examine if baseline levels of esophageal hypervigilance, fear of pain, and intolerance of uncertainty influence outcomes. Additionally, while we did find that threat led to significantly heightened skin conductance responses, 15/46 participants were excluded, which may have affected statistical power and the conclusions that can be drawn.

Although this study was mechanistic in nature and conducted in healthy volunteers, the findings have potential clinical relevance. Patients with esophageal disorders frequently anticipate aversive sensations, particularly in contexts that reliably provoke symptoms (e.g., eating or swallowing). Our results suggest that such anticipatory threat may heighten perceptual and emotional responses. Slower responding under threat in our paradigm may also point to difficulty disengaging attention from the esophageal sensations, though this interpretation remains speculative given the limitations of reaction time as a measure of attentional processes. This raises the possibility that anticipatory threat could help explain symptom exacerbation in everyday contexts. These findings highlight threat anticipation as a candidate mechanism whose clinical relevance and potential modifiability now require direct testing in patient populations, particularly to determine whether it contributes to symptom persistence or treatment response.

Strengths of this study include its ecologically valid design and the integration of multiple measures (perceptual, emotional, behavioral, and psychophysiological) to comprehensively capture responses to threat. The within‐subject, counterbalanced design enhanced sensitivity to detect threat effects while minimizing between‐subject variability. However, several limitations should be noted. First, the study sample consisted of healthy young adults and participants intolerant of the esophageal catheter were excluded, which may have created a tolerant subsample and limits generalizability to clinical populations. At the same time, participants hyposensitive to the electrical stimulation were also excluded, which may have created a subsample more sensitive to stimulation than the broader population. Second, while we were sufficiently powered to detect differences in our main analyses, the modest sample size reduced statistical power for exploratory analyses that required averaging across trials (e.g., mediation and moderation analyses). Third, while the electrical stimulation offers precise experimental control, it is likely a novel stimulus for most participants and may not be comparable to naturally occurring esophageal sensations (e.g., heartburn, dysphagia). Fourth, participants were recruited through the university participant pool and may have taken part in prior experimental studies; however, prior exposure to esophageal electrical stimulation was not systematically assessed and cannot be ruled out as a potential source of variability. Fifth, although the reinforcement ratio used in our paradigm was intended to limit the strength and frequency of cue‐pain associations, partial reinforcement schedules, even at relatively low rates, can support cue‐outcome learning and conditioned responding [43, 44]. As such, we cannot rule out that associate learning between the threat cue and the occasional painful outcomes contributed, in part, to the perceptual and physiological differences observed between our threat and control conditions. Finally, cue color was not counterbalanced across participants, such that the blue cue consistently signaled threat and the yellow cue consistently signaled control. As a result, cue color was confounded with experimental condition, and we cannot rule out that pre‐existing affective associations of differences in perceptual salience between the two colors contributed to the observed differences between conditions, particularly for fear ratings, which were made in direct response to the cue itself. Future iterations of this paradigm should counterbalance cue color across participants to isolate the effect of threat anticipation from potential color‐specific effects. Additionally, because cue onset was not independently marked as an event in our physiological recording, SCRs could only be time‐locked to the onset of the electrical stimulation rather than the cue itself. Given the short (1‐s) interval between cue and stimulation onset and the characteristic rise time of electrodermal responses, our design cannot fully disentangle anticipatory arousal to the threat cue from the physiological response to the stimulation. The elevated SCR observed during threat trials therefore likely reflects a combination of anticipatory and stimulus‐evoked arousal, rather than a pure measure of anticipatory threat responding. Future research should include larger and more diverse samples, examine patient populations, and employ additional indices of attentional engagement (e.g., pupil dilation or neural measures) to further elucidate the mechanisms underlying threat‐related perception.

In conclusion, this study provides insight into how the anticipation of threat to the esophagus can produce perceptual, emotional, behavioral, and psychophysiological changes. Together, these findings support the role of central processes in modulating symptom perception, even in the absence of changes in peripheral stimulation. Although further research is needed, particularly in clinical populations, these results highlight threat appraisal as a potentially modifiable target for interventions aimed at reducing esophageal symptom burden. In addition, this study provides the field with a novel experimental paradigm to allow ecologically valid testing in a patient population.

## Author Contributions

L.G., D.R., S.V.D., J.Z., and L.V.O. designed the research study. L.G. and J.W. performed the research. L.G., J.W., and L.V.O. analyzed the data. L.G. wrote the manuscript. J.W., D.R., S.V.D., J.Z., and L.V.O. provided critical review of the manuscript.

## Funding

This study was supported by a Junior Postdoctoral Fellowship of the Research Foundation Flanders (PI: LG; FWO, 12A7822N). Manuscript revision was supported by NIDDK K23DK149536 (L.G.).

## Conflicts of Interest

The authors declare no conflicts of interest.

## Supporting information


**Supplemental Figure 1:** Within‐subject mediation model examining fear as a mediator of the threat‐related increase in esophageal intensity.
**Supplemental Figure 2:** Within‐subject mediation model examining reaction time as a mediator of threat‐related changes in esophageal intensity.
**Supplemental Figure 3:** Distribution of ΔFear and ΔReaction Time (RT) scores.
**Supplemental Figure 4:** Distribution of Scores for Fear of Pain Questionnaire (FPQ‐III), Intolerance of Uncertainty (IUS‐12) and Esophageal Hypervigilance and Anxiety Scale (EHAS).

## Data Availability

The data that support the findings of this study are openly available in Github at https://github.com/labgas/proj_threat_esophagus.
