# Supplementary material for: Anticipated Threat Amplifies Esophageal Perception in the Absence of Peripheral Change
Source: Neurogastroenterol Motil. 2026 Nov 12;38(11):e70503. doi: 10.1111/nmo.70503 (PMC13756556; doi:10.1111/nmo.70503)
Supplement: Supplementary file 1 [file NMO-38-e70503-s001.docx]

**Supplementary File**

**METHODS**

**Patient Reported Outcome Measures**

Esophageal Hypervigilance and Anxiety Scale (EHAS). The EHAS^1^ is a 15-item self-report measure used to evaluate esophageal-specific hypervigilance (increased attention towards the esophagus and esophageal sensations) and anxiety (worry about symptoms and their potential consequences). The measure includes a total score, as well as a hypervigilance and anxiety subscale score. Each item has 5 response options ranging in value 0 “Strongly Disagree” to 4 “Strongly Agree”, yielding a total possible score ranging from 0 to 60. Higher scores indicate greater esophageal-specific hypervigilance and anxiety.

Fear of Pain Questionnaire – III (FPQ-III). The FPQ-III^2,3^ is a 30 item self-report measure assessing how fearful individuals are of experiencing pain associated with various experiences (e.g., breaking your arm, receiving an injection, getting a paper cut). Each item has 5 response options ranging from 1 “Not at All” to 5 “Extremely”, yielding a total possible score ranging from 5-150. Higher scores indicate greater fear of pain.

Intolerance of Uncertainty Scale – 12 item (IUS-12). The IUS-12^4,5^ is a 12-item measure assessing the construct intolerance of uncertainty, defined as an individual’s difficulty tolerating uncertain or ambiguous situations. Each item has 5 response options ranging from 1 “Not at All Characteristic of Me” to 5 “Entirely Characteristic of Me”, yielding a total possible score ranging from 5-60. Higher scores indicate greater intolerance of uncertainty.

**Skin Conductance Response**

Electrodermal activity was continuously recorded using the Biopac MP36R system and AcqKnowledge software (Biopac Systems Inc., Goleta, CA). Electrodermal activity was sampled at 1000 Hz and analyzed following the procedures outlined by Braithwaite et al. (2013).^6^

Phasic electrodermal activity was derived using a 0.5 Hz high-pass filter and a 5-second baseline estimation window from the stimulus. Skin conductance responses (SCRs) are defined as the difference between baseline and peak in window linked to stimulus onset. Thus, SCRs were event-locked to the onset of the electrical stimulation, not the cue (i.e., blue/yellow rectangle signifying the condition). Cue onset was not independently marked as an event in the physiological recording, so an anticipatory response window locked specifically to cue onset could not be scored separately from the stimulus-evoked response. They were identified using an amplitude threshold of 0.03 μS, and a rejection rate of 10% per guidelines proposed by Braithwaite et al. (2013). ^6^ Participants who did not exhibit SCRs exceeding this threshold in at least half of trials were classified as non-responders and excluded from SCR analyses. Individual SCRs were event-locked and labeled according to trial type (Threat vs. Control). Mean SCR amplitude for each condition was log-transformed to reduce skewness and used in subsequent statistical analyses.

**Thresholding Procedure**

The thresholding procedure began at 2 mA and increased in 4 mA increments until the stimulation was rated as *“faint discomfort”* (i.e., one categorical level above the target rating of *“sensation”* on the 9-point categorical scale). The intensity was then decreased in 2 mA steps until the participant rated the stimulus as *“sensation.”* Once this point was reached, the intensity was again increased in 2 mA steps until the participant rated it as *“faint discomfort”* or higher. This allowed us to identify the highest intensity rated as *“sensation.”*

Next, the intensity was increased in 4 mA steps until the participant reported *“strong pain”* (i.e., one categorical level above the target rating of *“pain, yet tolerable”* on the 9-point categorical scale) or until the maximum allowable intensity was reached (60 mA for the esophagus; 99 mA for the hand). The intensity was then decreased in 2 mA steps until the participant rated the stimulus as *“pain, yet tolerable.”* Finally, the intensity was again increased in 2 mA steps until *“strong pain”* or the maximum intensity limit was reached.

The average of the two highest intensities rated as *“sensation”* and *“pain, yet tolerable”* was calculated separately for the esophageal and hand stimulations and used as the non-painful and painful stimulation intensities, respectively, in the experimental task.

**Intensity Check**

During this check, participants received one non-painful and one painful stimulus in random order and rated their intensity using the 9-point categorical scale described previously. Stimulus intensities were adjusted if necessary (i.e., if the non-painful stimulus was rated above or below “sensation,” or the painful stimulus was rated above or below “pain, yet tolerable”). This was done through increasing/decreasing the stimulus intensity by 2mA until the desired rating was reached.

**Exploratory Statistical analyses**

*Mediation Analyses*

The Psychobiological Model of Disorders of Gut-Brain Interaction^13^ proposes that heightened perceived threat elicits fear, which in turn increases interoceptive threat monitoring (attention) and visceral perception (Figure 1). Guided by this model, we conducted within-subject mediation analyses to examine whether the effect of the threat condition on intensity ratings was mediated by reaction time or fear ratings. Reaction time, intensity ratings, and fear ratings were averaged across all trials for the esophageal blocks. Models were estimated in which the delta value (threat – control) for intensity served as the outcome, and the delta values for reaction time and fear ratings were entered as mediators.

*Moderation Analyses*

Moderation analyses examined whether baseline self-reported psychological processes (esophageal hypervigilance, intolerance of uncertainty, and fear of pain) influenced the effect of the threat condition on intensity and fear ratings. These variables were selected based on the hypothesis that individuals with greater esophageal hypervigilance or fear of pain, or lower tolerance of uncertainty, would exhibit amplified responses to anticipated threat, in line with the Psychobiological Model.^13^ Specifically, we tested whether lower tolerance of uncertainty, greater esophageal hypervigilance, and/or greater fear of pain were associated with larger threat–control differences in intensity and fear ratings. Moderation analyses were conducted using the MEMORE macro (version 2.1) for SAS, with delta scores (threat-control) for intensity and fear ratings entered as dependent variables and baseline questionnaire scores entered as moderators.

*Skin Conductance Response*

Finally*,* we assessed differences in physiological arousal (i.e., skin conductance response) between experimental conditions. Specifically, we conducted exploratory analyses mirroring the main analyses of whether the threat condition would elicit greater arousal than the control condition, esophageal stimulation would elicit greater arousal than hand stimulation, and the effect of the threat condition might vary by location. A linear mixed-effects model examined the effects of condition (threat vs. control), body location (esophagus vs. hand), and their interaction (condition by location). Block included as a fixed-effect covariate to adjust for habituation. A random intercept for each participant was included to account for repeated observations. Skin conductance response amplitudes were log-transformed to reduce skewness.

**RESULTS**

**Skin Conductance Response**

Fifteen participants were excluded due to either being non-responders (N=13), missing measurements (e.g., equipment malfunction; N=1), or excessive artifacts that prevented reliable analysis (N=1). Therefore, the final sample included 31 participants. A linear mixed-effects model revealed a significant main effect of the threat condition, such that responses were higher during threat trials compared to control trials (β = 0.078, SE = 0.021, p < .001). There was no significant main effect of location (β = 0.013, SE = 0.021, p = 0.52) or condition by location interaction (β = 0.015, SE = 0.021, p = 0.46), indicating that the effect of threat did not differ between esophageal and hand stimulation. There was a trend toward an effect of block (β = –0.079, SE = 0.041, p = 0.05), suggesting a possible decrease in magnitude across the session, which is expected with SCR.

**Relationships between Reaction Time, Fear, and Intensity**

We used mediation and moderation analyses to explore the relationships between threat, reaction time, and intensity and fear ratings within the esophageal location. First, we tested whether the effect of the threat condition on intensity ratings was mediated through fear and/or reaction time. As expected, threat was significantly associated with an increase in esophageal intensity ratings (total effect: β = 6.01, p < .001, 95% CI [4.26, 7.76]) in both the fear and reaction time models. For the fear model, threat was significantly associated with increased fear ratings (β = 23.38, p < .001, 95% CI [17.56, 29.20]). However, fear was not significantly associated with intensity while controlling for threat (β = 0.09, p = 0.20, 95% CI [-0.05, 0.24]) and the indirect effect of threat on intensity via fear was not significant (indirect effect: β = 2.22, 95% CI [−0.27, 5.39]), indicating that changes in fear did not mediate the impact of threat on esophageal intensity perception (Supplemental Figure 1). For the reaction time model, threat did not significantly influence reaction time (β = 0.01, p = 0.36, 95% CI [-0.02, 0.05]), and changes in reaction time did not predict changes in intensity while controlling for threat (β = -2.05, p = 0.81, 95% CI [-19.14, 15.04]). The indirect effect of threat on intensity through reaction time was not significant (indirect effect: β = -0.03, 95% CI [−0.74, 0.20]), indicating that reaction time did not mediate the impact of threat on esophageal intensity perception (Supplemental Figure 2).

Next, we tested whether baseline esophageal hypervigilance and anxiety, intolerance of uncertainty, or fear of pain moderated the effect of threat on intensity and fear ratings. Specifically, we were interested in whether lower tolerance of uncertainty, greater esophageal hypervigilance/anxiety, and/or greater fear of pain were associated with larger threat–control differences in intensity and fear ratings. None of the variables moderated the effect of threat on intensity (esophageal hypervigilance/anxiety: β = -0.02, p = 0.73, 95% CI [-0.19, 0.13]; IUS: β = -0.09, p = 0.42, 95% CI [-0.33, 0.14]; FOP: β = -0.03, p = 0.61, 95% CI [-0.14, 0.08]) or fear (hypervigilance/anxiety: β = -0.07, p = 0.79, 95% CI [-0.61, 0.46]; intolerance of uncertainty: β = -0.20, p = 0.61, 95% CI [-0.99, 0.59]; fear of pain: β = 0.24, p = 0.19, 95% CI [-0.12, 0.59]). Notably, intolerance of uncertainty (β = 0.71, p < 0.001, 95% CI [0.31, 1.11]) was associated with greater fear in the control condition, and fear of pain was associated with greater fear in both the threat (β = 0.45, p = .05, 95% CI [-0.01, 0.92]) and control conditions (β = 0.22, p = 0.03, 95% CI [0.02, 0.42]).

**Supplemental Figure 1. Within-subject mediation model examining fear as a mediator of the threat-related increase in esophageal intensity.**

**Supplemental Figure 2. Within-subject mediation model examining reaction time as a mediator of threat-related changes in esophageal intensity.**

**Supplemental Figure 3. Distribution of ΔFear and ΔReaction Time (RT) scores**

**Supplemental Figure 4. Distribution of Scores for Fear of Pain Questionnaire (FPQ-III), Intolerance of Uncertainty (IUS-12) and Esophageal Hypervigilance and Anxiety Scale (EHAS)**

**References**

1.	Taft TH, Triggs JR, Carlson DA, et al. Validation of the oesophageal hypervigilance and anxiety scale for chronic oesophageal disease. Aliment Pharmacol Ther 2018;47(9):1270-1277. (In eng). DOI: 10.1111/apt.14605.

2.	McNeil DW, Rainwater AJ, 3rd. Development of the Fear of Pain Questionnaire--III. J Behav Med 1998;21(4):389-410. (In eng). DOI: 10.1023/a:1018782831217.

3.	van Wijk AJ, Hoogstraten J. Dutch translation of the Fear of Pain Questionnaire: factor structure, reliability and validity. Eur J Pain 2006;10(6):479-86. (In eng). DOI: 10.1016/j.ejpain.2005.06.008.

4.	Carleton RN, Norton MA, Asmundson GJ. Fearing the unknown: a short version of the Intolerance of Uncertainty Scale. J Anxiety Disord 2007;21(1):105-17. (In eng). DOI: 10.1016/j.janxdis.2006.03.014.

5.	Helsen K, Van den Bussche E, Vlaeyen JW, Goubert L. Confirmatory factor analysis of the Dutch Intolerance of Uncertainty Scale: Comparison of the full and short version. J Behav Ther Exp Psychiatry 2013;44(1):21-9. (In eng). DOI: 10.1016/j.jbtep.2012.07.004.

6.	Braithwaite JJ, Watson DG, Jones R, Rowe M. A guide for analysing electrodermal activity (EDA) & skin conductance responses (SCRs) for psychological experiments. Psychophysiology 2013;49(1):1017-1034.

7.	Kroenke K, Spitzer RL, Williams JB. The PHQ-9: validity of a brief depression severity measure. J Gen Intern Med 2001;16(9):606-613. (In eng). DOI: 10.1046/j.1525-1497.2001.016009606.x.

8.	Spitzer RL, Kroenke K, Williams JB, Löwe B. A brief measure for assessing generalized anxiety disorder: the GAD-7. Arch Intern Med 2006;166(10):1092-7. (In eng). DOI: 10.1001/archinte.166.10.1092.
